# Ready-to-wear strain sensing gloves for human motion sensing

**DOI:** 10.1016/j.isci.2021.102525

**Published:** 2021-05-08

**Authors:** Sara S. Mechael, Yunyun Wu, Yiting Chen, Tricia Breen Carmichael

**Affiliations:** 1 Department of Chemistry and Biochemistry, University of Windsor, Windsor, Ontario N9B 3P4, Canada

**Keywords:** Sensor, Bioelectronics, Electronic engineering, Nanotechnology fabrication

## Abstract

Integrating soft sensors with wearable platforms is critical for sensor-based human augmentation, yet the fabrication of wearable sensors integrated into ready-to-wear platforms remains underdeveloped. Disposable gloves are an ideal substrate for wearable sensors that map hand-specific gestures. Here, we use solution-based metallization to prepare resistive sensing arrays directly on off-the-shelf nitrile butadiene rubber (NBR) gloves. The NBR glove acts as the wearable platform while its surface roughness enhances the sensitivity of the overlying sensing array. The NBR sensors have a sheet resistance of 3.1 ± 0.6 Ω/sq and a large linear working range (two linear regions ≤70%). When stretched, the rough NBR substrate facilitates microcrack formation in the overlying metal, enabling high gauge factors (62 up to 40% strain, 246 from 45 - 70% strain) that are unprecedented for metal film sensors. We apply the sensing array to dynamically monitor gestures for gesture differentiation and robotic control.

## Highlights

• Sensing arrays are prepared on ready-to-wear nitrile butadiene rubber (NBR) gloves
• Solution-based deposition is used to pattern gold sensing arrays on the NBR surface
• The roughness-enhanced sensitivity offers high gauge factors (62-246) to 70% strain
• Motion sensing is demonstrated for gesture differentiation and robotic control

## Graphical Abstract

**Figure:**
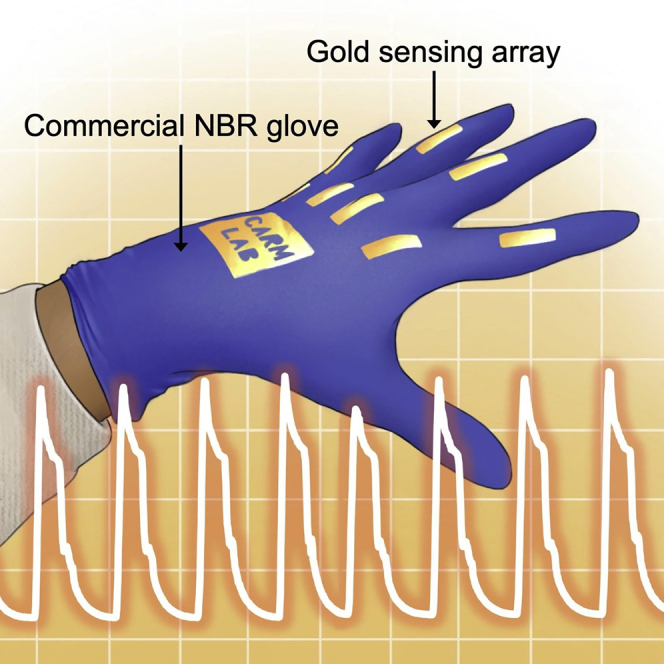

## Introduction

The human body comprises thousands of biological, biochemical, and physiological processes, each a data set relating to human health and well-being (Sanctis and Loreti, 2017). Although humans can have a qualitative awareness of some of these processes, quantitative data are inaccessible to our perception. Augmenting our bodies with wearable sensors can provide routine access to personalized biometric data to empower individuals to make data-informed behavioral adjustments in daily life to improve health and fitness or to employ this information for contemporary applications such as virtual reality or robotic control. Access to data such as heart rate, brain activity, muscular response, or glucose levels will create a feedback system between individuals and wearables, forming the next evolutionary step toward sensor-based human augmentation. Today's commercial wearable sensors, available in formats such as wristbands or goggles, have popularized the everyday collection of personalized data and are an important step in this evolutionary process. Although these devices are designed to be worn on the human body, their bulky and rigid components are a mismatch with the softness of the human body (Asokan et al., 2016; Lu et al., 2014; O'Flynn et al., 2013). In response to this limitation, the research community has developed new sensing materials integrated with elastomers that can conform to the surfaces of the human body, providing the intimate contact necessary for reliable and unobtrusive data collection (Amjadi et al., 2016). However, there has been far less attention to incorporating sensing materials into wearable formats designed to be worn on the human body. Integrating these new soft sensors into wearable formats is pivotal to the evolution of sensor-based human augmentation. In particular, human hands are evolutionarily critical, exhibiting great versatility of motion to perform a wide range of tasks from basic grasping of objects to more complex functions such as playing rapid sequences of piano keys, wielding a paintbrush or pen, or communicating using sign language. Wearable sensors that detect and monitor hand gestures can provide data to quantify these motions (Wang et al., 2020a); in addition, augmentation with these sensors opens up new opportunities such as assessing range of motion for therapeutic treatments, interpreting gestures like sign language or piano playing for transcription, and interfacing with virtual reality. Here, we unite functionality and wearability in a resistive thin-film sensing array fabricated directly on the surface of an off-the-shelf laboratory disposable glove. Disposable gloves are made to fit the complex contours of the human hand, making them an ideal substrate for sensors to map hand-specific tasks.

Progress in the development of soft and conformable strain sensors has used conductive materials including metal nanowires (Amjadi et al., 2014; Gong et al., 2015), carbon nanotubes (Ryu et al.,; Yamada et al., 2011), metal films (Filiatrault et al., 2015), graphene (Li et al., 2016; Wang et al., 2014), liquid metal (Chen et al., 2020; Gao et al., 2019; Li and Lee, 2017), and ionic liquid (Choi et al., 2017; Chossat et al., 2015; Zhang et al., 2017) on stretchable substrates such as siloxane elastomers (Ryu et al.,; Wang et al., 2018), latex (Gong et al., 2015), and polyurethane (Ding et al., 2016; Jeon et al., 2017). Strain sensors can employ resistive, capacitive, or optical sensing mechanisms, where a change in one of these properties can be correlated to strain (Souri et al., 2020). Resistive strain sensors incorporating solid conductive sensing materials undergo physical changes to their microstructure or nanostructure with strain, such as disconnection of nanostructured materials or crack formation in conductive films, which result in measurable resistance changes with strain. Among these material candidates, metal films deposited on elastomers are especially appealing due to the ease of fabrication by physical vapor deposition or solution-based metallization methods. The mechanical mismatch between the elastomeric substrate and metal film results in the generation of cracks that increase in density, length, and width perpendicular to the strain direction and proportional to strain, resulting in an increase in the resistance of the metal film (Li et al., 2012; Xiang et al., 2005). The sensitivity of metal film strain sensors is the magnitude of this resistance change with strain. When the strain is removed, the elastomeric substrate facilitates the return to its original dimensions, rejoining crack edges and decreasing the resistance of the metal film, enabling repetitive use of the sensors (Li et al., 2012). The simplest metal-on-elastomer strain sensor, a planar gold film deposited by physical vapour deposition (PVD) on polydimethylsiloxane (PDMS), undergoes a large resistance change (>100%) with high sensitivity but only remains conductive to ∼22% strain due to fatal channel cracking. Channel cracks are wide cracks that initiate at defect sites and quickly propagate across the film, cutting off all conductive pathways (Lacour et al., 2003). The useful sensing range for wearable electronics, however, must encompass the maximum strain exerted by bending joints such as fingers and elbows, which is ∼35%–60% (Amjadi et al., 2015; Boland et al., 2014; Yan et al., 2014). Despite the high sensitivity offered by the cracking mechanism, the working range of these sensors often falls short of the useful sensing range for wearable electronics. Depositing metals on rough elastomer surfaces has been used as a strain-engineering strategy to improve the working range of metal/elastomer strain sensors (Filiatrault et al., 2015; Lambricht et al., 2013; Lee et al., 2016). Surface roughness suppresses channel crack formation by concentrating strain in distributed valleys and promoting microcrack formation whose propagation is impeded by topographical peaks, thereby facilitating the formation of a reticular cracking pattern that preserves conductivity to high elongations (Lee et al., 2016; Xu et al., 2011). Roughness can be achieved by casting elastomeric prepolymers on rough surfaces to introduce topography to the elastomer surface (Lambricht et al., 2013; Lee et al., 2016) or by depositing rough polymeric layers as an interlayer between the elastomeric substrate and metal coating (Filiatrault et al., 2015). Wearable strain sensors made by depositing a thin gold film on a rough poly(vinyl acetate) glue interlayer on PDMS exhibited a useful working range to ∼50%; however, the sensor was affixed to a finger using a bandaid and tape for testing (Filiatrault et al., 2015).

In fact, it is often the case that laboratory prototypes affix soft strain sensors to the body by taping or gluing onto clothing (Boland et al., 2014; Gong et al., 2015; Yamada et al., 2011) and disposable laboratory gloves (Cai et al., 2013; Dong et al., 2020; Lee et al., 2014; Lee et al., 2015; Qu et al., 2020; Ryu et al.,) or directly on skin (Amjadi et al., 2015; Choi et al., 2017; Filiatrault et al., 2015; Wang et al., 2014; Zhang et al., 2016) in locations including hands (Filiatrault et al., 2015), wrists (Wang et al., 2014), eyelids (Lee et al., 2017a), and throat (Park et al., 2015) to monitor large-scale muscle (Gong et al., 2015) and joint (Lee et al., 2015) movements, as well as human physiological signals such as pulse (Gong et al., 2015) and heart rate (Chou et al., 2016). The responsibility of affixing soft sensors or complex sensing arrays to a garment or to the body should not lie with the user; instead, the evolution of sensor-based human augmentation demands the seamless integration of sensors into ready-to-wear garments and other wearable articles. Moreover, since this integration itself can affect or limit the mechanical behavior of the sensor, it is important to investigate the wearable device as a whole. One approach to solve this problem uses a full fabrication method in which the sensing material and wearable article are fabricated together, enabling excellent integration between the two. Examples of this approach include the fabrication of polymeric glove-like platforms equipped with finger caps or Velcro closures to attach to the hand (Chossat et al., 2015; Kim et al., 2019; Muth et al., 2014). As part of the fabrication, active sensing materials are often either embedded in the polymer or encapsulated in microfluidic channels within the polymer. These wearable sensors have been used to detect the motions of the human hand through changes in resistance of the embedded or encapsulated sensing material. This full fabrication approach provides wearable devices that are customized to fit a specific user's hand, with potential utility in health care and physiotherapy in the same way orthopedics is customized. This level of specific customization is not necessary, however, for many applications outside of health care and also presents challenges for mass production of low-cost and potentially disposable wearable sensors.

Here, we reduce the fabrication complexity of the full fabrication approach by fabricating a sensor array directly on off-the-shelf disposable nitrile butadiene rubber (NBR) laboratory gloves. We preserve the wearability of the NBR glove by chemically activating its surface for the selective solution-based deposition of a patterned array of strain-sensing gold films on the hand joint locations. The ready-to-wear electroless nickel immersion gold (ENIG)/NBR sensing array developed in this work aims to simplify the user experience by addressing the challenge of complex sensing array application onto the body while also demonstrating that the garment itself can act as an impactful component of the device. The intrinsic roughness of the commercial NBR glove facilitates the cracking mechanism of the gold sensors, where microcrack formation and propagation causes an increase in resistance correlated to the strain. In this way, each sensor on the glove contributes to a map of information that collectively describes the position of the wearer's hand. We demonstrate the application of this information for gesture differentiation and robotic control, in which the resistance data from the sensor array are translated into English alphabet letters or robotic commands, respectively.

## Results and discussion

### Fabrication of patterned ENIG sensors on NBR gloves

We used commercially available, disposable lab gloves made from NBR, a copolymer of acrylonitrile and butadiene, as the substrate for our wearable strain sensors. Using electron beam deposition to deposit gold coatings on the surface of an NBR lab glove produced metal coatings that appear inhomogeneous and populated with pinhole defects in scanning electron microscopy (SEM) images (Figure S1) and exhibit high sheet resistance (1.0 ± 0.4 MΩ/sq). The incompatibility of NBR with PVD is similar to that reported in previous reports of metal deposition on hydrocarbon-based polymeric and elastomeric substrates using PVD, which causes damage and produces non-conductive films (Ohring, 2002; Vohra et al., 2018). Therefore, we instead deposited conductive gold coatings on NBR lab gloves using an additive solution-based metallization process called the ENIG plating method, which we have previously reported to coat PDMS and synthetic textile substrates with conformal gold films (Chen et al., 2019; Wu et al., 2018, 2020). The advantages of this low-cost ENIG method include established industrialized processes used routinely in printed circuit board fabrication, relatively low deposition temperatures, and scalability. The ENIG process uses two solution-based plating steps, beginning with the electroless deposition (ELD) of a nickel film followed by an immersion gold process (Chen et al., 2019; Liu et al., 2007). In the nickel ELD process, a palladium-tin colloidal catalyst adsorbed on the substrate surface catalyzes the reduction of Ni^2+^ ions in the plating solution to metallic nickel. Nickel deposition occurs autocatalytically thereafter as a reducing agent in the plating solution is consumed. Subsequent immersion of the nickel-coated substrate in a potassium gold cyanide solution results in a galvanic displacement reaction in which Ni atoms in the film reduce Au^+^ ions from solution, forming a metallic gold film on the surface and releasing Ni^2+^ ions into solution.

**Figure 1:**
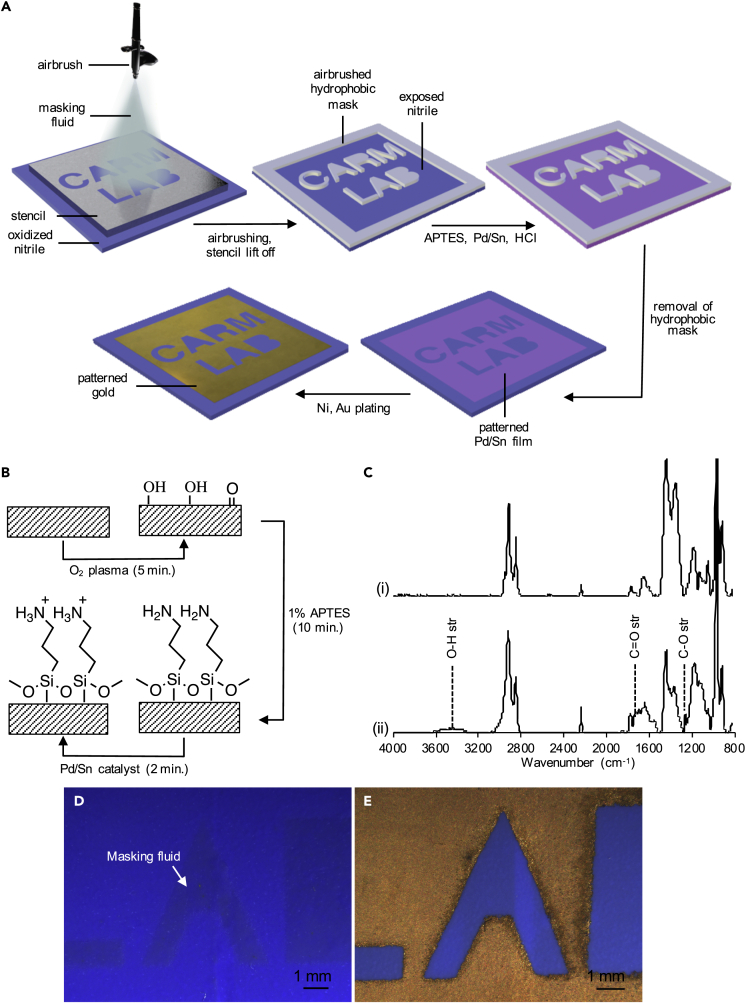
Fabrication of patterned ENIG sensors on NBR gloves (A) Schematic depicting patterned ENIG deposition on NBR. (B) Activation of NBR by plasma oxidation, reaction with APTES, and protonation of the amine groups. (C) ATR-FTIR spectra of the NBR surface before (i) and after (ii) oxygen plasma treatment. (D) Stereomicrographs of a patterned masking fluid film and (E) the resulting patterned ENIG film. See also Figure S3 for gold adhesion.

We developed a method for patterned ENIG deposition on NBR lab gloves by spray coating a resist pattern through a stencil mask to direct the adsorption of catalytic Pd/Sn colloids on the surface (Figure 1A). The process begins with an initial plasma oxidation step to both increase the wettability of the NBR surface and create oxidized functional groups for subsequent chemical surface modification (Figure 1B). Plasma oxidation for 5 min reduced the water contact angle from 30 ± 11° to <10°. Attenuated total reflection-Fourier transform infrared (ATR-FTIR) spectroscopy of the NBR surface shows that the native NBR surface comprises the expected hydrocarbon peaks including C-H stretches at 2918 and 2848 cm-^1^, a C=C stretch at 1641 cm^−1^, C-H deformation at 1437 cm^−1^, and an alkene = C-H stretch at 966 cm^−1^, as well as the nitrile C≡N stretch at 2239 cm^−1^ (Figure 1C(i)). Plasma oxidation of NBR adds a heterogeneous mixture of oxidized functional groups to the surface, indicated by peaks due to -OH, C=O, and C-O stretches at 3464 cm^−1^, 1729 cm^−1^, and 1261 cm^−1^, respectively (Figure 1C(ii)). We patterned the oxidized NBR surface by spray coating a masking fluid, an aqueous latex solution commonly used as a resist in watercolor painting, through a stainless steel stencil mask. The masking fluid contacts and wets the NBR surface through the openings in the stencil mask and then dries to form a patterned hydrophobic resist film (Figure 1D). The areas not covered by the masking fluid remain accessible to chemical modification to facilitate the binding of Pd/Sn colloids, thus enabling the selective activation of the NBR surface for nickel ELD. Pd/Sn colloids consist of a Pd-rich core, a hydrolyzed Sn^2+^/Sn^4+^ shell, and associated chloride ions that give the colloids a negatively charged surface that can electrostatically bind to cationic functional groups. We prepared the accessible regions of the patterned NBR substrate for Pd/Sn binding by immersion in a solution of 3-aminopropyltriethoxysilane (APTES) to create a patterned amino-terminated surface (Figure 1B). We confirmed functionalization by APTES using ATR-FTIR spectroscopy (Figure S2, Table S1) (Celina et al., 1998; Gunasekaran et al., 2007; Kawashima and Ogawa, 2005; Miller et al., 2008; Zhou et al., 2007). Immersing the APTES-patterned NBR in an acidic solution of Pd/Sn colloids protonates the amino groups to form positively charged ammonium groups that electrostatically bind the Pd/Sn colloids. Etching the Sn shell in a 6 M HCl solution exposes the catalytic palladium core, thus activating the surface for nickel ELD. After peeling away the patterned masking fluid layer with tweezers, immersion in the nickel ELD solution deposits a metallic nickel film; subsequent galvanic displacement in the immersion gold solution results in the formation of patterned gold films within the activated regions of the surface pattern (Figure 1E). The resulting patterned gold films exhibit excellent adhesion to the NBR surface and pass the tape test without transfer of gold to the tape (Figure S3).

### Characterization of patterned ENIG films on NBR

**Figure 2:**
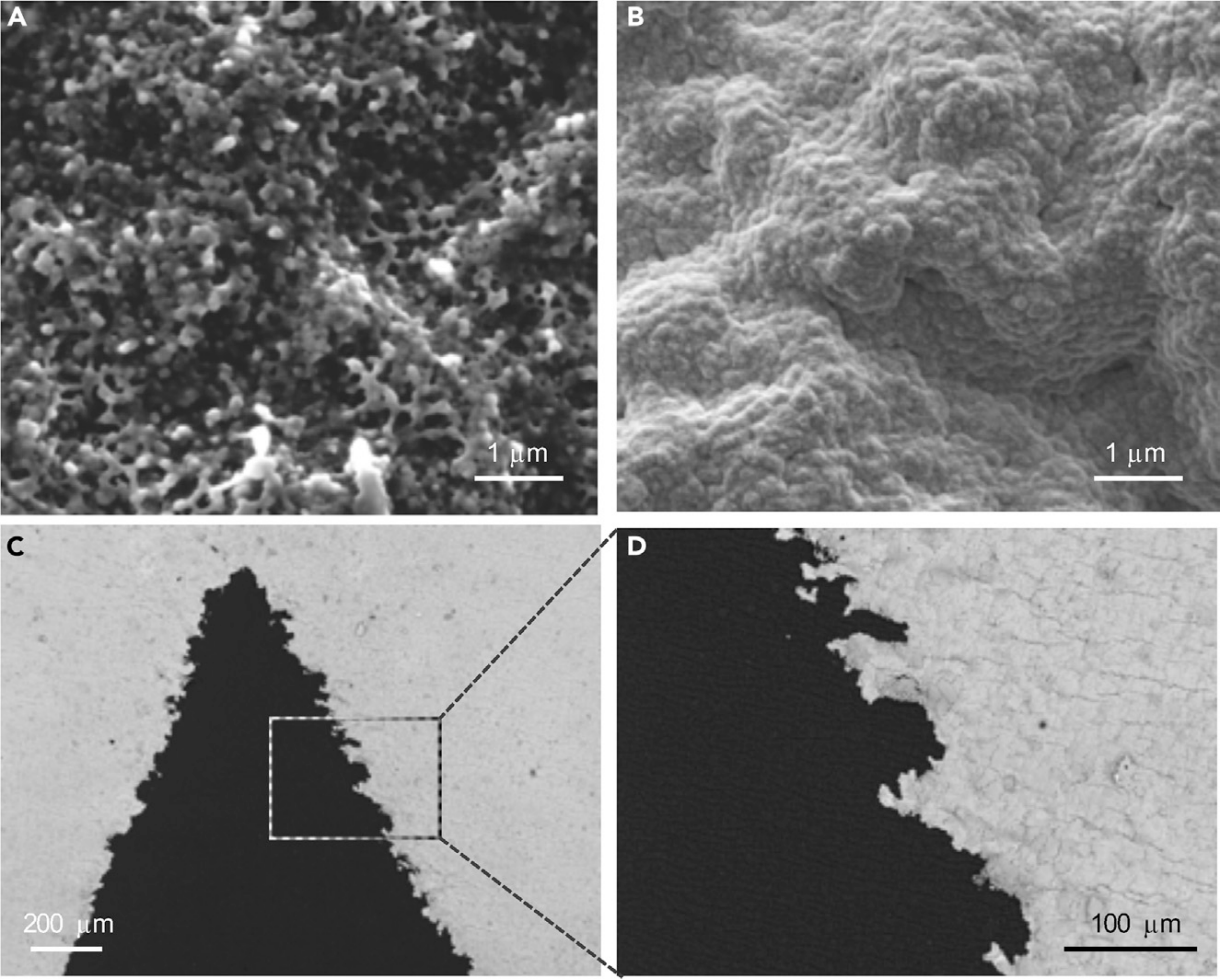
Characterization of patterned ENIG films on NBR (A) SEM images of the oxidized NBR surface; (B) ENIG film on NBR; (C and D) Patterned ENIG line edges. See also Figures S4–S6 for further surface characterization.

The ENIG process deposits a continuous and conductive gold coating over the NBR surface. Native NBR presents a rough surface (Figure 2A) with an average root mean square (RMS) roughness of 278 ± 40 nm (Figure S4A). Plasma oxidation slightly changes the morphology of the surface, reducing the RMS roughness to 179 ± 4 nm (Figure S4B). The ENIG process conformally coats these topographical features with gold (Figure 2B), resulting in an RMS roughness of 270 ± 30 nm (Figure S4C). Analysis of the ENIG film using energy-dispersive X-ray spectroscopy (EDS) detected gold as well as a small amount of residual nickel (<1 wt %) in the film (Figure S5). The thickness of the ENIG films measured using cross-sectional SEM was ∼82 nm (Figure S6). The sheet resistance of the film was 3.1 ± 0.6 Ω/sq, slightly higher than that of a gold film of the same thickness prepared by e-beam evaporation on a smooth glass substrate (0.9 ± 0.1 Ω/sq). The higher sheet resistance of ENIG on NBR can be attributed to the higher roughness of the NBR substrate compared to glass (Blair, 1981; Timoshevskii et al., 2008). The patterning resolution was limited by pinning of air bubbles by the hydrophobic mask edges during metallization, limiting the line width that we could successfully print to 0.3 cm. SEM furthermore revealed that patterned ENIG films possess a rough line edge, with an RMS line edge roughness of 22 ± 8 μm (Figures 2C and 2D). This roughness arises from seeping or wicking of the masking fluid under the edges of the stencil mask during spray coating due to the intrinsic roughness of the NBR surface that affects the stencil-substrate contact, as well as small variation in the directionality of the manual spray coating. Although the pattern resolution and line edge roughness may be improved by addressing these processing challenges, for example, by the addition of mechanical agitation during the solution metallization process to dislodge bubbles, the current process is satisfactory for patterning the mm-scale features necessary for wearable strain sensing applications.

### Characterization of ENIG/NBR strain sensors

**Figure 3:**
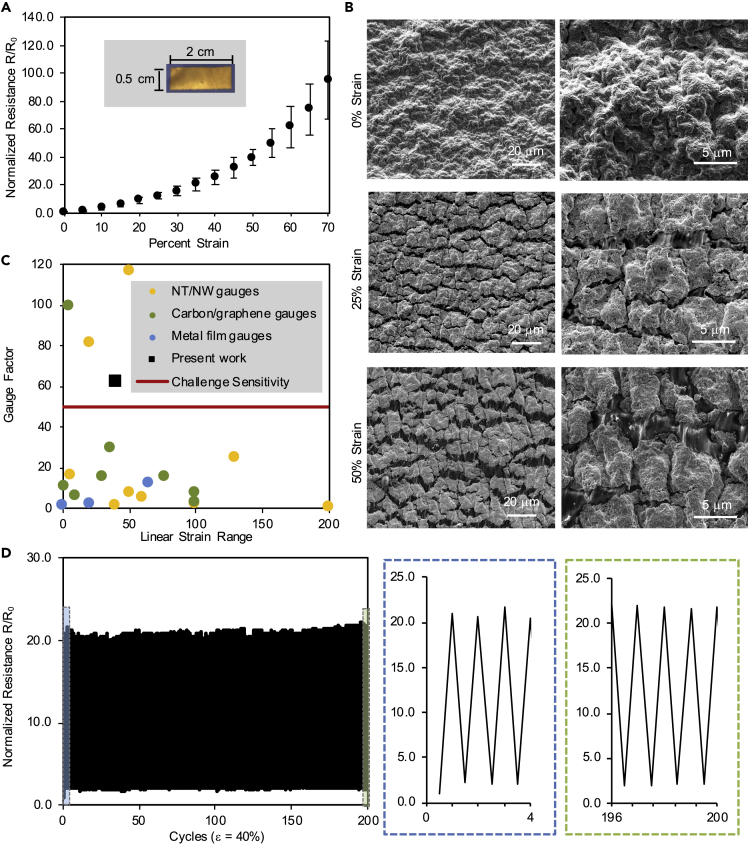
Characterization of ENIG/NBR strain sensors (A) Normalized change in resistance as a function of percent strain of the ENIG/NBR sensors. Data points represent mean ± standard deviation. (B) SEM images showing crack propagation through the ENIG film at 0%, 25%, and 50% strain. Stretching direction is vertical. (C) Comparison of gauge factors and linear strain ranges for published research on metal nanowire and carbon or ZnO nanotube-based strain gauges (yellow circles) (Amjadi et al., 2014; Amjadi et al., 2015; Gong et al., 2015; Li et al., 2017; Ryu et al.,; Xiao et al., 2011; Yamada et al., 2011; Yu et al., 2017), carbon black, and graphene-based gauges (green circles) (Dong et al., 2020; Jeong et al., 2015; Kong et al., 2014; Li et al., 2016; Qu et al., 2020; Yan et al., 2014), metal film gauges (blue circles) (Filiatrault et al., 2015; Kang et al., 2015; Lee et al., 2014, 2017b), and the present work on NBR-based gold films (black square). (D) Normalized change in resistance of ENIG/NBR strain sensors over 200 cycles of 40% strain. Right insets show the first four cycles (blue) and the last four cycles (green). See also Figures S7–S12 for further sensor characterization.

The rough surface of the NBR substrate causes the formation of short, jagged cracks in the overlying ENIG film that increases the resistance with strain while still preserving electrical percolation pathways to elongations of 70%. We fabricated ENIG strain sensors on NBR with dimensions of 2.0 cm × 0.5 cm (Figure 3A inset) and characterized the resistance change of these sensors with strain (Figure 3A). The initial resistance of the sensors (8 ± 3 Ω) increased with elongation due to the formation of cracks in the ENIG film, reaching ∼85 times the initial resistance at 70% strain. Adjusting the aspect ratios of the sensors does not significantly impact the resistance change with strain (Figure S7). SEM imaging at 0, 25, and 50% strain revealed the nature of the cracks that cause the resistance increase: Cracks <100 μm in length form in the ENIG film along the valleys of the NBR surface topography. Metal films deposited on rough surfaces are known to initiate cracks in valleys that propagate until they reach a peak in the topography, where propagation is impeded (Lee et al., 2016; Xu et al., 2011). Cracks in the ENIG/NBR sensors widen from ∼1.5 μm in width at 25% strain to ∼2.5 μm in width at 50% strain (Figure 3B).

Several important parameters influence the suitability of the ENIG/NBR resistive sensors for motion detection, including stretchability, the linearity of the strain response, sensitivity or gauge factor (GF), and durability (Amjadi et al., 2016). The stretchability of ENIG/NBR strain sensors, which remain conductive to 70% strain, is more than sufficient to detect the maximum strain exhibited by the proximal interphalangeal (PIP) and metacarpophalangeal (MCP) joints of the human hand, which are reported to have a maximum strain of 35%–45% (Amjadi et al., 2015; Boland et al., 2014; Yan et al., 2014). The resistance response of the sensor to strain can be fitted with two linear regions. The first linear region is defined as being between 0% and 40% strain with a correlation coefficient (R^2^) of 0.96 (Figure S8). The second linear region encompasses 45%–70% strain with a R^2^ of 0.97 (Figure S8). The working range of the sensors for gesture sensing therefore primarily falls within the first linear region. A linear relationship between resistance change and strain is important for strain sensors, as nonlinearity complicates sensor calibration. The sensitivity of ENIG/NBR strain sensors can be quantified by computing the GF over the working range. The GF is defined as the change in resistance in response to strain, where R is resistance, R_0_ is initial resistance, and ε is the mechanical strain (Mechael et al., 2018):

$$\text{GF}=\left(\left(\text{R}-{\text{R}}_{0}\right)/{\text{R}}_{0}\right)/\varepsilon$$

The ENIG/NBR strain sensors demonstrate high sensitivity with a GF of 62 between 0% and 40% strain and a GF of 246 between 45% and 70% strain. A 2016 review of soft strain sensors by Amjadi et al. noted the trade-off that occurs between high sensitivity and high linearity during stretching and defined a challenge sensitivity of GF > 50 over the linear strain range (Amjadi et al., 2016). The high sensitivity of ENIG/NBR strain sensors surpasses this challenge sensitivity, exceeding the sensitivity of other reported metal film-elastomer gauges by more than 500% (Figure 3C). Additionally, our sensors achieve a high GF in comparison to most other reported carbon-based gauges and nanowire- or nanotube-based composites. The increasing variation in normalized resistance with increasing strains is a common behavior of cracking-based strain sensors. Within the expected working range of 0–45% strain, the ENIG/NBR sensors exhibit low variation and well-resolved resistances. Therefore, the ENIG/NBR sensors demonstrate excellent strain detection resolution of ±5% strain at 45% strain.

The recovery time of the sensors during cyclic loading is limited by the viscoelastic strain recovery of the NBR glove substrate, which requires time to regain its original dimensions after a load is removed (Figure S9A), consequently delaying the reconnection of the overlying gold during recovery. We used dynamic mechanical analysis to characterize this strain recovery behavior of the NBR substrate and demonstrated that the recovery time to reach 90% of the initial length depends on the duration of loading (Figures S9B and S9C). After a 2 min loading duration, the NBR takes 5.7 min to recover to 90% of its initial length, whereas after a 2 s loading duration, the NBR takes 3 s to recover. We subjected ENIG/NBR strain sensors to repetitive cycles of 40% strain with sufficient strain recovery time in between cycles and measured the resistance in the relaxed (0%) and strained (40%) states (Figure 3D). The sensors exhibited normalized resistances at 40% strain that remained consistent throughout 200 cycles. The normalized resistance at 40% strain on the first and 200^th^ cycle was 20.9 and 21.9, respectively. Optical micrographs of these sensors also showed negligible change in the surface after the first and 200^th^ cycle (Figure S10). We did not observe electrical or mechanical failure after 200 cycles. Repeating this experiment with no relaxation time through repetitive, uninterrupted 0–40% strain cycles causes an upward creep in the normalized resistance in the relaxed state due to insufficient relaxation time of the viscoelastic substrate. Although the normalized resistance at the maximum strain (40%) remains within one standard deviation of the expected normalized resistance, upon unloading, there is insufficient time for the sensor to recover, resulting in an upward creep of the normalized resistance in the relaxed state (0%) (Figure S11). This behavior implies that the repeated opening of cracks in the ENIG film under 40% strain is consistent over 200 cycles, and it is the viscoelasticity of the NBR substrate that causes the upward creep during repeated unloading cycles.

We investigated the durability of the ENIG/NBR strain sensors to physical handling by poking and scratching the gold coating with tweezers, which resulted in a minimal resistance increase (normalized resistance <2) (Video S1). The application of pressure to the sensor surface similarly has a minimal effect on the resistance, where light pressure (comparable to touching a phone screen) to extreme pressure (the highest pressure that can be exerted using a thumb) applied to the surface results in a normalized resistance ranging from 1.2 to 3.6 (Video S2). We also investigated the temperature sensitivity of the ENIG strain sensors. The resistance of the sensors remained stable to temperatures up to 100°C (Figure S12A), and the change in resistance with stretching to 70% elongation at 100°C was also within one standard deviation of sensors characterized at room temperature (Figure S12B).

Video S1. Durability of ENIG/NBR sensors to abrasion, related to STAR Method s

Video S2. Pressure insensitivity of the ENIG/NBR sensors, related to STAR Method s

### Wearable ENIG/NBR strain sensors

**Figure 4:**
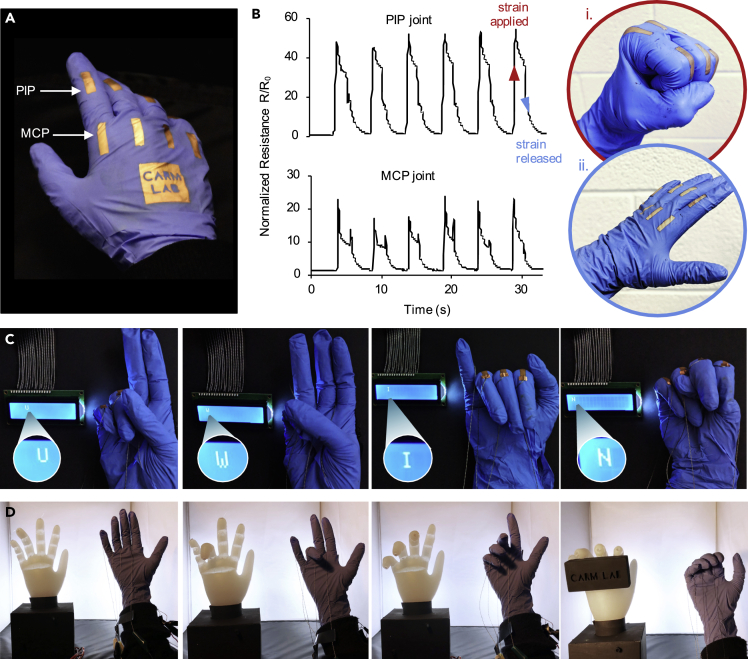
Wearable ENIG/NBR strain sensors (A) A photograph of ENIG/NBR sensor array. (B) Normalized resistance change during repetitive finger bending cycles of sensors on PIP and MCP joints. Insets show (i) the strained state, and (ii) the released state. (C and D) (C) The application of the ENIG/NBR sensing array for gesture differentiation and (D) for robotic control. See also Figures S13–S17.

We used an array of ENIG/NBR sensors directly patterned on a glove to detect and map the motions of the fingers. We designed a glove with 2.0 cm × 0.5 cm sensors located at each position of the fingers' PIP and MCP joints (Figure 4A) and studied the responsiveness of the sensors by measuring the resistance change as the wearer opens and closes their hand (Figure 4B). Sensors located at both PIP and MCP joints were responsive to the gestures, indicated by an increase in the normalized resistance in the strained state (Figure 4B(i)) and a decrease in the relaxed state (Figure 4B(ii)). At the onset of strain, the ENIG/NBR sensors have a short response time of ∼0.5 s (Figure S13), which was measured as the time it takes to reach the maximum resistance. The onset of strain triggers a brief (∼0.5 s) and small overshoot of ∼10x the initial resistance that is due to the viscoelastic stress-relaxation behavior of the NBR substrate (Figure S14). This is a common behavior of resistive strain sensors, and the overshoot duration is comparable to that of other strain sensors implemented for motion monitoring, which have been reported to fall between <1s and 10 s (Liu et al., 2021; Nankali et al., 2020; Wang et al., 2020a, 2020b). After stabilizing, the sensor response corresponds to a strain of ∼45% on the PIP joint and ∼20% on the MCP joint. As the hand opens and strain is released, the normalized resistance falls as the sensor recovers. Although the recovery time of the NBR substrate is dictated by the strain recovery behavior of the NBR, in the practical demonstration of the ENIG/NBR sensors monitoring finger movements during cyclical bending, the short loading time of ∼2 s leads to a ∼3 s recovery time to reach 90% of the initial resistance (Figure S13) (Koncar, 2019), which enables utility in real-time sensing.

The linear working range, high sensitivity, and durability of the soft sensing array equip the glove with the qualities necessary to collect real-time data from the movement of the wearer's hands. We demonstrated this dynamic sensing of the strain sensing glove by integrating it in a circuit that collects voltage information and uses an algorithm to differentiate between a small library of four American sign language (ASL) alphabet gestures (Figure S15, Data S1). To establish a map of the wearer's hand gesture, the circuit uses a voltage divider setup, where a known resistor is placed in series with one of the ENIG/NBR sensors, which acts as a variable resistor. A voltage of 5 V is split along the path proportionally to the resistance difference between the known resistor and ENIG/NBR sensor. By measuring the voltage at the known resistor, the algorithm calculates the voltage and resistance at the ENIG/NBR sensor. The algorithm then takes the combination of resistance inputs from each finger and compares it to a bank of conditions of various ASL gestures. The algorithm is able to correctly identify gestures made by the wearer and then displays the corresponding English alphabet letter on a liquid crystal display. In Figure 4C, the wearer uses ASL to sign “U,” “W,” “I,” and “N,” an abbreviation for the University of Windsor, and the algorithm correctly displays the English alphabet letter on the liquid crystal display.

Another way to use the data map generated by the glove sensors is to link the real-time motions of the fingers to a robotic replica. In this application, we used the strain sensing glove as a robotic hand controller by preparing a circuit and algorithm to map voltage data collected from the NBR sensor array and used it to operate the fingers in a fabricated robotic hand (Figure S16, Data S2). We fabricated the robotic hand by casting the elastomer Ecoflex into a glove and subsequently hollowing channels along the length of each finger where fishing line could be run through (Figures S17A–S17C). Each finger had a fishing line anchored to the fingertips and tautly connected to a servo motor in the base of the model. If the wearer bends their fingers, the algorithm prompts the servo motor to rotate, which consequently pulls the fishing line on the corresponding robotic finger. The downward pull on the fishing line forces the Ecoflex finger to collapse at cuts made in the joint locations, effectively bending the robotic joints (Figure S17D). We again used a voltage divider setup to create a map of the wearer's gestures. Since this setup uses a 5 V source, the voltage measured at the known resistor must be between 0 and 5 V and depends on the resistance of the ENIG/NBR sensor connected in series. Therefore, we map this voltage range to the servo motor angle of 0-180°, meaning a voltage reading of 0 V corresponds to a servo motor angle of 0°, while a voltage reading of 5 V prompts the servo motor to rotate 180°. By mapping the voltage range to the servo motor angle range, we are able to use the entire bending range of the finger to partially or completely bend the robotic joints. It is thus possible for the wearer to control the movement of the robotic hand simply by performing the gesture (Figure 4D, Video S3).

Video S3. Dynamic robotic control using the ENIG/NBR glove sensing array, related to figure 4

Integrating sensors with wearable platforms is critical to the evolution of comfortable sensor-based human augmentation. To the best of our knowledge, the present work is one of the first examples of direct functionalization of a ready-to-wear glove garment for wearable strain sensors. The ready-to-wear ENIG/NBR sensing array developed in this work aims to simplify the user experience by addressing the challenge of complex sensing array application onto the body, while also demonstrating that the garment itself can act as an impactful component of the device. We developed a prototype of this next-generation ready-to-wear ENIG/NBR sensing array by seamlessly integrating metallic thin-film sensor arrays on the surfaces of commercial laboratory gloves, providing a simple fabrication approach with great potential for mass production, contributing to a future of ubiquitous, low-cost, and even disposable wearable sensors. NBR gloves are a convenient, readily accessible vehicle to provide intimate contact between the human hand and metallic sensor array, enabling real-time mapping of human motion. The NBR gloves not only provide a readily wearable platform but also contribute to the functionality of the strain sensor due to the roughness of the NBR surface, which facilitates the formation of a fine microcracking network in response to strain. The resulting high GFs are unprecedented for metal film strain sensors and competitive with carbon- and nanomaterial-based sensors. This scalable, low-cost, additive fabrication ENIG process is promising for patterning motion sensing arrays, and future work will explore alternative commercially available substrates that can minimize viscoelastic response, as well as application-specific array designs that generate data-rich outputs to differentiate between large libraries of complex gestures such as ASL. The integration of strain-insensitive wiring or wireless communication systems is another promising way to advance this work toward comfortable sensor-based human augmentation.

### Limitations of the study

This work features the deposition of a sensing array on a commercial ready-to-wear glove, which demonstrates the ability to functionalize everyday garments for true integration of soft sensors on wearable platforms. However, the use of any commercial product in a sensing device is accompanied by fixed material properties that become integrated in the final device performance. In this work, we fabricate a sensing array on commercial NBR gloves and observe that the surface roughness of the glove enhances the sensitivity of the overlying sensors. We also demonstrate that the recovery time of these sensors is dictated by the viscoelastic properties of NBR and that the recovery time is dependent on the duration of the applied strain. Therefore, we acknowledge that longer strain durations than what were presented in this work may influence the recovery time in dynamic applications. We have performed mechanical analysis to show that this limitation in recovery time is directly correlated to the strain recovery behavior of the commercial NBR substrate. This highlights a need for more suitable wearable platform materials for fully integrated wearable sensors.

Additionally, wiring the ENIG/NBR strain sensor to a power source limits the wearability of the sensing glove, which is an ongoing challenge for the entire field of wearable electronics. In the demonstrations of the ENIG/NBR sensing glove, we used conductive thread instead of bulky insulated wires to connect the sensors to a power supply; however, contacts to the sensors were made using liquid metal and copper tape, which are not ideal for real-world use. Moreover, the power supply and circuit for the demonstrations must also be incorporated into wearable designs for real-world use of wearable sensors. This challenge has inspired a global effort to develop soft wiring or wireless solutions.

We also note that the spray coating method used to pattern a resist for metallization is responsible for the resulting line edge roughness of the metallized patterns. Process improvements such as automating the spray coating to maintain a constant spray angle and duration could improve this resolution; however, the resolution reported in this work is sufficient for the macroscale of the patterned sensor features.

## STAR★Methods

### Key resources table

**Table:** 

REAGENT or RESOURCE	SOURCE	IDENTIFIER
**Chemicals**		

3-aminopropyltriethoxysilane (APTES)	Sigma-Aldrich	440140, CAS 919-30-2
Cataposit 44 Catalyst Concentrate	Dow	Cataposit44
Cataprep 404 Concentrate	Dow	Cataprep404
hydrochloric acid (HCl)	Fisher Chemical	CAS 7647-01-0
nickel (II) sulfate hexahydrate	Oakwood Chemical	CAS 10101-97-0
sodium pyrophosphate decahydrate	Sigma-Aldrich	221368, CAD 13472-36-1
dimethylamine borane (DMAB)	Oakwood Chemical	CAS 74-94-2
Gobright TAM-55	Uyemura	TAM-55-R, TAM-55-M10, AURUNA 6700-Au Salts
poly(octadecenyl-alt-maleic anhydride) (POMA)	Sigma-Aldrich	776866
Art Masking fluid	Winsor & Newton	Winsor & Newton Art Masking Fluid
Eutectic gallium-indium (EGaIn)	Sigma-Aldrich	495425
Ecoflex-30 Kit	Smooth-On	SO# 86340A, 86340B

**Software and algorithms**		

ImageJ	Rasband, 1997-2018	https://imagej.nih.gov/ij/
Analyze_Stripes	Bickford (2013)	https://imagejdocu.tudor.lu/doku.php?id=macro:analyze_stripes
Arduino code for differentiation of “U,” “W,” “I,” and “N” ASL gestures	This work	
Arduino code for dynamic robotic hand control	This work	

### Resource availability

#### Lead contact

Further information and requests for resources and reagents should be directed to and will be fulfilled by the lead contact, Tricia Breen Carmichael (tbcarmic@uwindsor.ca).

#### Materials availability

This study did not generate new unique reagents.

#### Data and code availability

The Arduino codes created for the gesture differentiation and robotic control demonstrations are available as supplemental data. See Data S1 and Data S2 files.

### Method details

VWR Soft Nitrile Examination Gloves were obtained from VWR. All other chemicals were purchased commercially and used as received.

#### E-Beam deposition of gold on NBR

NBR samples were exposed to 1500 mTorr oxygen plasma (Harrick Plasma, PDC FMG) for 5 minutes (power of 10.2 W, standard cubic centimeters per minute of 63 mL/min). A 30 Å titanium (Ti) adhesion layer and 820 Å of gold (Au) was deposited using e-beam evaporation (Figure S1B). A deposition rate of 4 Å/s and 5 Å/s was maintained for Ti and Au, respectively.

#### Electroless-nickel immersion-gold (ENIG) on NBR

The NBR surface was exposed to oxygen plasma for 5 min, and then sequentially immersed in a 1% (v/v) solution of APTES in deionized water for 10 min, a Pd/Sn solution (prepared from Cataposit 44 and Cataprep 404 [Dow], as directed by the manufacturer) for 2 min, and a 6 M HCl solution for 1 min with water rinsing and nitrogen drying after removal from each solution. The NBR was then metallized in a nickel ELD solution (0.08 M NiSO_4_·6H_2_O, 0.14 M Na_4_P_2_O_7_·10H_2_O, and 0.07 M dimethylamine borane in water) for 10 min with sonication. After rinsing with water, the Ni-coated NBR gloves were immersed in an immersion gold solution (Gobright TAM-55, Uyemura) at 60°C for 40 min with stirring.

#### Verification of APTES Modification by ATR-FTIR-Detection of POMA Tag

We used chemical tagging and ATR-FTIR to verify the presence of APTES on plasma-oxidized NBR (Figure S2). Since the primary amine surface generated after APTES modification is challenging to detect spectroscopically, we tagged the APTES-modified NBR surface with poly(octadecenyl-alt-maleic anhydride) (POMA) to generate detectable amide and carboxylic acid stretches. To deposit APTES and tag the surface with POMA, we first activated the NBR surface in 1500 mTorr oxygen plasma for 5 min, and then immersed the sample in a solution of 1% (v/v) APTES in water for 10 min. After rinsing in de-ionized water and drying with nitrogen, a 1% (v/v) POMA solution in water was dropcast on the sample surface and left for 1 min. The sample was rinsed with water and sonicated in water for 10 min to remove physisorbed POMA. ATR-FTIR analysis reveals the amide I stretch (1714 cm^-1^), the amide II stretch (1573 cm^-1^), and carboxylic acid stretches (1779 cm^-1^ and 1222 cm^-1^), consistent with the presence of POMA (Figure S2 and Table S1).

#### Patterning ENIG on NBR

To prepare small samples of ENIG/NBR sensors, two adjacent strips of double-sided tape were applied to the NBR glove and cut out using scissors. The NBR was then adhered to a glass slide to keep the NBR substrate flat during airbrushing. Commercially available artists' masking fluid (Winsor & Newton) was airbrushed (Neo for Iwata CN Gravity Feed Airbrush, 0.35 mm nozzle) through a stainless-steel stencil onto the NBR surface. The stainless-steel stencil was quickly removed, and the masking fluid left to air dry. The hydrophobic mask and double-sided tape were peeled off before immersion in the Ni ELD solution. The sample was then clipped to a glass slide for immersion in the Ni ELD and Au baths.

#### Wearable sensor fabrication

The PIP and MCP joint locations on the NBR glove were flattened against glass slides and masking fluid was airbrushed over 2 cm x 0.5 cm masks. The glass slides were removed and the NBR glove was folded into a compact sample that exposed the joint locations, which was held in place by adhesion between contacting surfaces covered by the hydrophobic mask. The hydrophobic mask was peeled off before immersion in the Ni ELD solution. The NBR glove was folded and clipped into a compact sample for immersion in the Ni ELD and Au baths. Contacts for electrical testing between the NBR sensing array and Arduino (Arduino Uno) circuit or Keithley 2601A Sourcemeter were made by taping down conductive thread with copper tape. A small drop of eutectic-gallium indium (EGaIn) was spread on the adhesive side of the copper tape to improve electrical contact with the conductive thread and ENIG/NBR sensors.

#### Characterization

ATR FT-IR spectra were collected using a Bruker IFS 66/v spectrometer equipped with a DGTS detector and Harrick Autoseagull accessory. Water contact angles were measured using the sessile drop method on a Rame-Hart contact angle goniometer. Two readings from at least three samples were averaged. Sheet resistance was measured on 1 cm^2^ samples, and resistance was measured on 0.5 cm x 2 cm samples. Averaged resistance and sheet resistance data shows readings for at least three samples. Samples were stretched using a micro-vice stretcher (S.T. Japan, USA, Inc) and resistance was measured during stretching at 5% increments of the sample's initial length using a Keithley 2601A Sourcemeter. Resistance was measured during bending cycles of the patterned glove while applying a 0.5 V voltage using the Keithley sourcemeter. Cycles of 40% strain were applied with 5 min rest between cycles and measured at 0% and 40% strain using a Keithley sourcemeter. Durability to abrasion and pressure was tested by monitoring the resistance using a Keithley sourcemeter while poking and scratching the sensors (Video S1), or applying pressure with a thumb (Video S2). Stereomicrographs were collected using a Leica M205 stereoscope. Atomic Force Microscopy (AFM) images were collected using a Digital Instruments Multimode atomic force microscope in tapping mode. A Veeco FESP cantilever with a tip of 8 nm radius and force constant of 2.8 N/m were used. Roughness measurements were determined using WSxM 5.0 Develop 7.0 software and the reported roughnesses are the average of at least three values. Scanning electron microscope (SEM) images and EDS spectra were collected using a Quanta 200 FEG Environmental Scanning Electron Microscope (SEM). Line-edge roughness of the patterned sensor was obtained by analyzing SEM images using the Analyze_Stripes macro (Bickford, 2013) for ImageJ (Rasband, 1997-2018). The reported line-edge roughness is an average of RMS line-edge roughness values from 6 images. Stress relaxation of the NBR substrate was characterized by dynamic mechanical analysis using a DMA850 (TA Instruments). NBR samples were cut to 2 cm x 1 cm and preconditioned with a 0.05 N load.

#### Sign language gesture differentiation

The glove was connected to an Arduino Uno microcontroller where the resistance was measured at each joint and an algorithm matched the English language letter to the electrical conditions required (Figure S15, Data S1). A voltage of 5 V is split between one known resistor and our glove resistor, sending the current from the glove resistor to an analog pin where an analog-to-digital converter converts the voltage (from 0-5 V) to a digital value (0-1023). The resistance is calculated based on the voltage and the program loops through an if-else program to determine which letter is being gestured by the wearer. To simplify the program, only the letters U, W, I, and N are defined and possible. The code then prints the letter to the LCD screen.

#### Fabrication of an Ecoflex robotic hand

We fabricated the robotic hand by casting Ecoflex 30 prepolymer (Smooth-on, 1:1 w/w ratio of Parts A:B) into a small NBR glove (Figure S17A). After the Ecoflex cured in the glove mold, we cut and peeled away the glove to obtain the hand-shaped Ecoflex model (Figure S17B). We cut wedge-shaped slits at the inner joint positions on the palm side of the hand model, such that the fingers could “bend” by hinging closed at the cut. A scalpel was used to hollow out channels parallel to the fingers. The channels extend from each finger down to the wrist. Syringe needle sheaths were cut to size and used to line the channels. Fishing line was threaded through each channel and anchored to the finger tips by tying a large bead that could not fit into the channel (Figure S17C). The opposite side of the fishing line was connected to a servo motor in a 0° position. The servo motors were fixed in a cardboard base that also holds the robotic hand upright. The servo motors were then connected to an Arduino with a program loaded to control the servo motor position. When the servo motors are commanded to rotate 180°, the fishing line is pulled down, forcing the Ecoflex finger to collapse at cuts made in the joint locations, effectively bending the robotic joints (Figure S17D).

#### Robotic hand controller

The glove was connected to an Arduino Uno microcontroller to control the joints in a soft robotic hand (Figure S16). A voltage of 5 V is split between one known resistor and our glove resistor, sending the current from the glove resistor to an anolog pin where an analog-to-digital converter converts the voltage (from 0-5 V) to a digital value (0-1023). The digital range is mapped to an angle range (0-180°) of a servo motor. The program in Data S2 loops every 15 ms, updating the servo position, and consequentially, the robotic hand's joint position.

### Quantification and statistical analysis

All averaged datasets including Figures 3A, S7, and S8, along with reported values of contact angle, sheet resistance, roughness, and line-edge roughness, are averages of at least 3 samples and are reported as mean ± standard deviation. See also method details for the number of samples tested in each experiment.

## Supplemental information

Document S1. Figures S1–S17 and Table S1

Data S1. Arduino code for differentiation of “U,” “W,” “I,” and “N” ASL gestures, related to figure 4 and STAR Methods

Data S2. Arduino code for dynamic robotic hand control, related to figure 4 and STAR Methods
