# Supplementary material for: Ready-to-wear strain sensing gloves for human motion sensing
Source: iScience. 2021 May 8;24(6):102525. doi: 10.1016/j.isci.2021.102525 (PMC8192569; doi:10.1016/j.isci.2021.102525)
Supplement: Document S1. Figures S1–S17 and Table S1 [file mmc1.pdf]

iScience, Volume *24*

Supplemental information

Ready-to-wear strain sensing gloves

for human motion sensing

Sara S. Mechael, Yunyun Wu, Yiting Chen, and Tricia Breen Carmichael

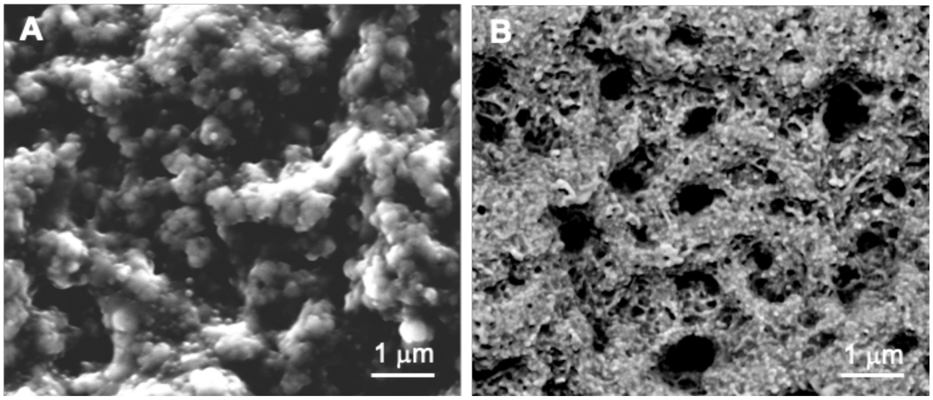

Figure S1. SEM images of **(A)** the native NBR surface, and **(B)** the NBR surface after e-beam deposition of 30 Å Ti and 820 Å Au. Related to STAR methods.

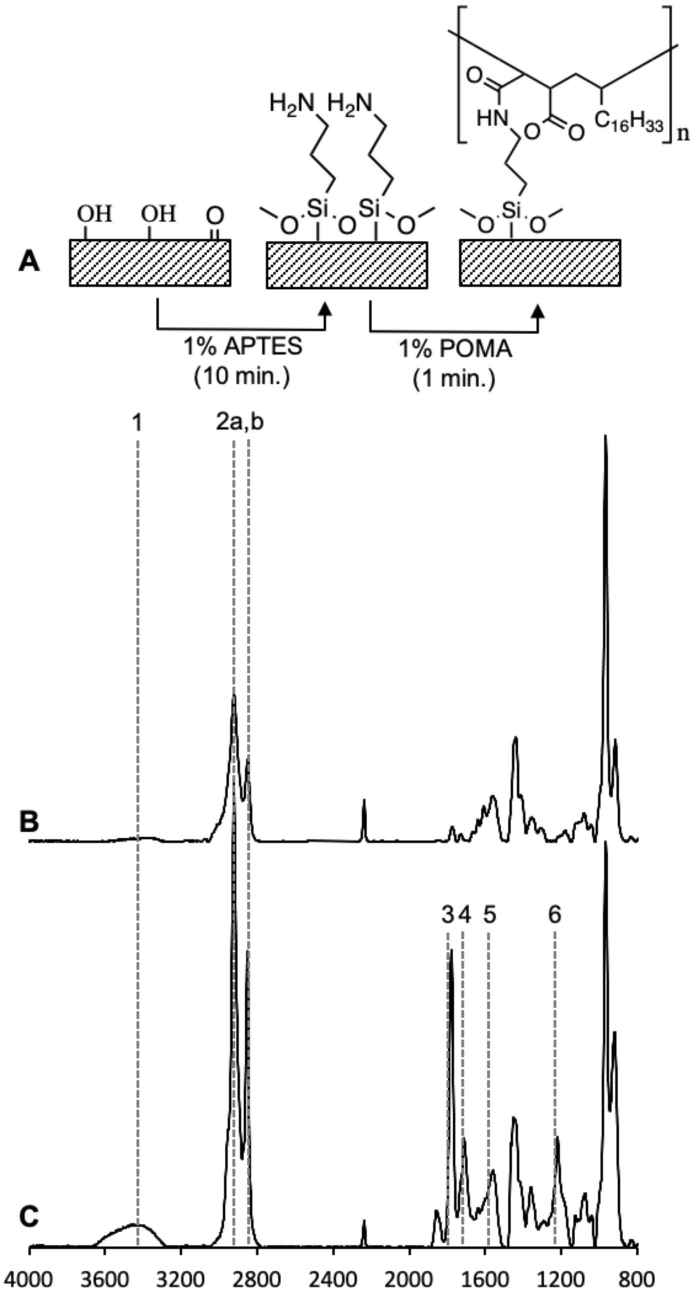

Figure S2. Verification of APTES modification by ATR-FTIR-detection of POMA tag. **(A)** Scheme depicting the reaction of oxidized NBR with 1% APTES followed by labeling the APTES with POMA for IR detection. ATR FT-IR spectra of **(B)** NBR after 5 min oxygen plasma exposure followed by 10 min treatment with 1% APTES in water and 10 min. sonication in water, and **(C)** after subsequent drop-casting of 1% POMA in water for 1 min. and 10 min. sonication in water. Number labels refer to peak assignments in Table S1. Related to STAR methods.

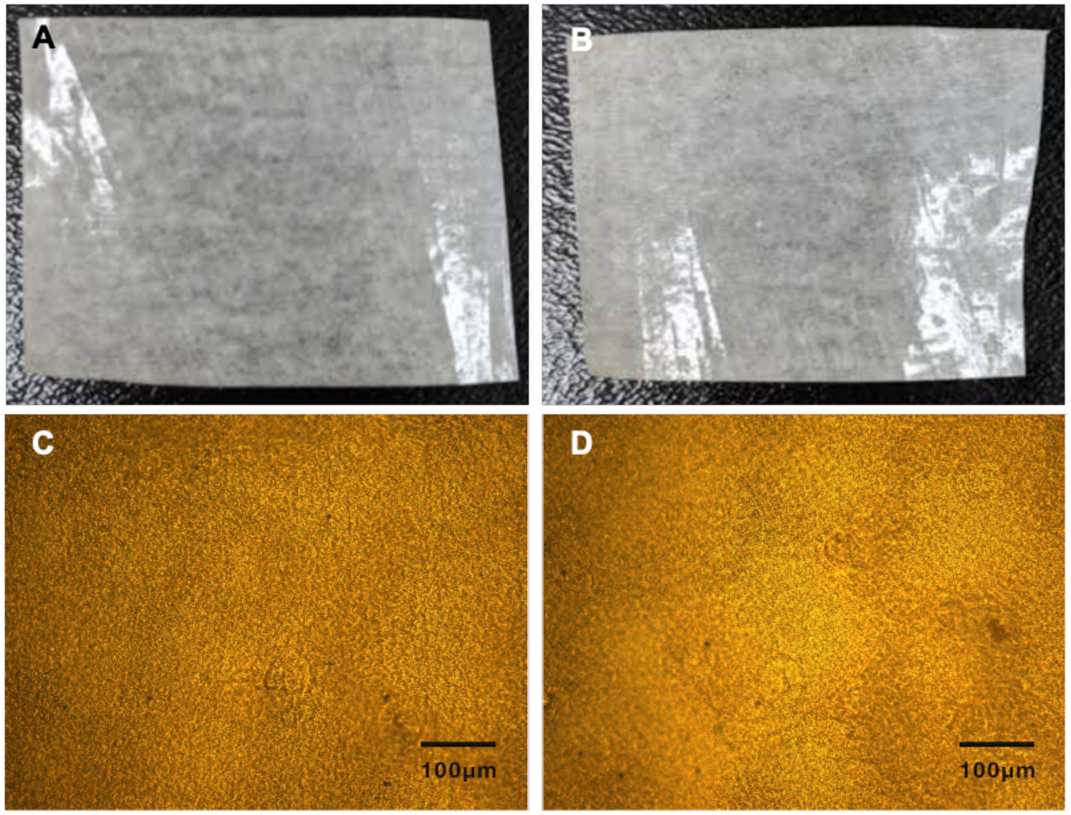

Figure S3. Gold adhesion test. Tape **(A)** before and **(B)** after lamination and delamination from the gold coatings on NBR. Optical micrographs of the gold surface on NBR **(C)** before and **(D)** after lamination and delamination of tape on the surface. Related to Figure 1.

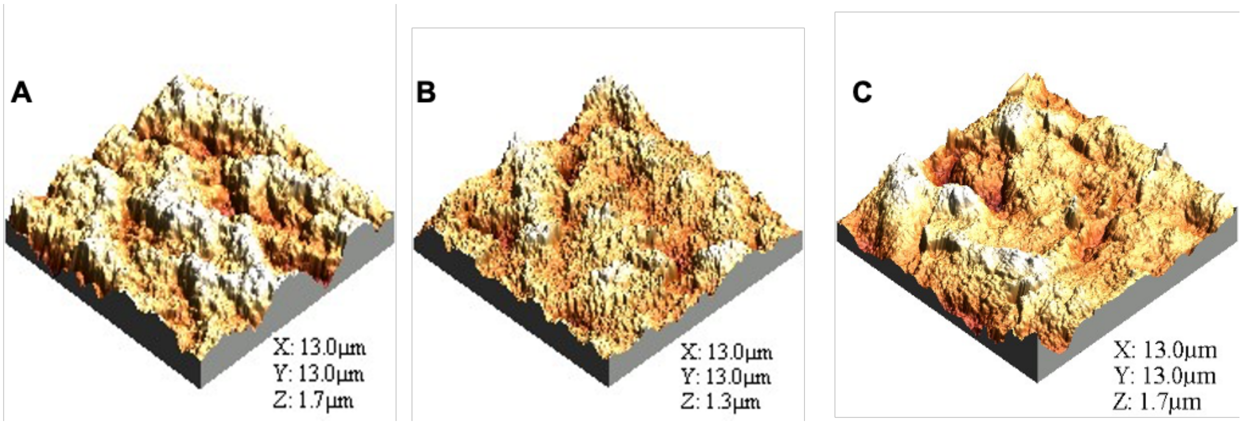

Figure S4. Atomic force micrographs of **(A)** the native NBR surface, **(B)** the oxidized NBR surface, and **(C)** the sensor surface after ENIG deposition. Related to Figure 2.

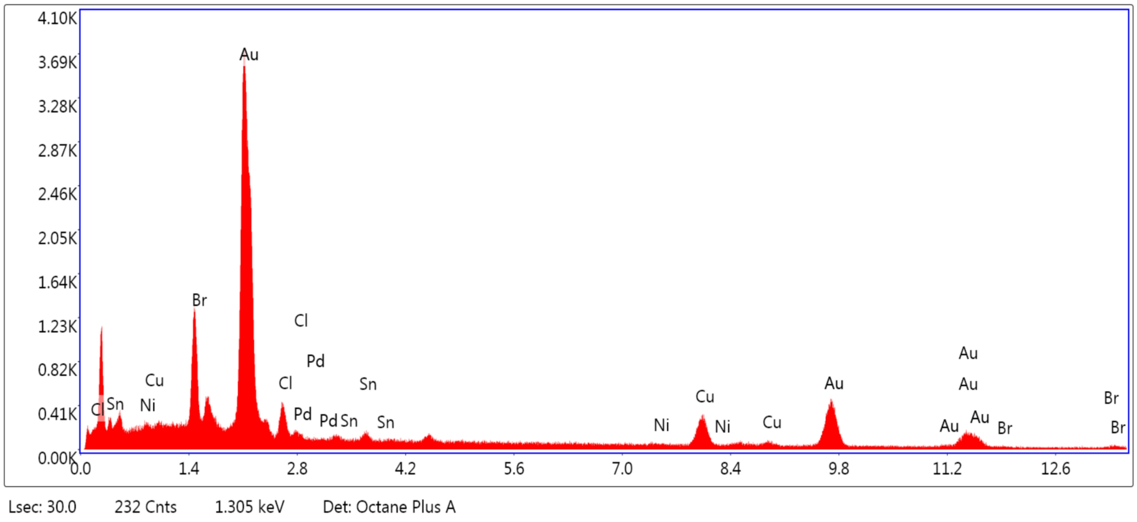

Figure S5. EDS spectrum of ENIG Au on NBR after 40 min in immersion gold solution. Related to Figure 2.

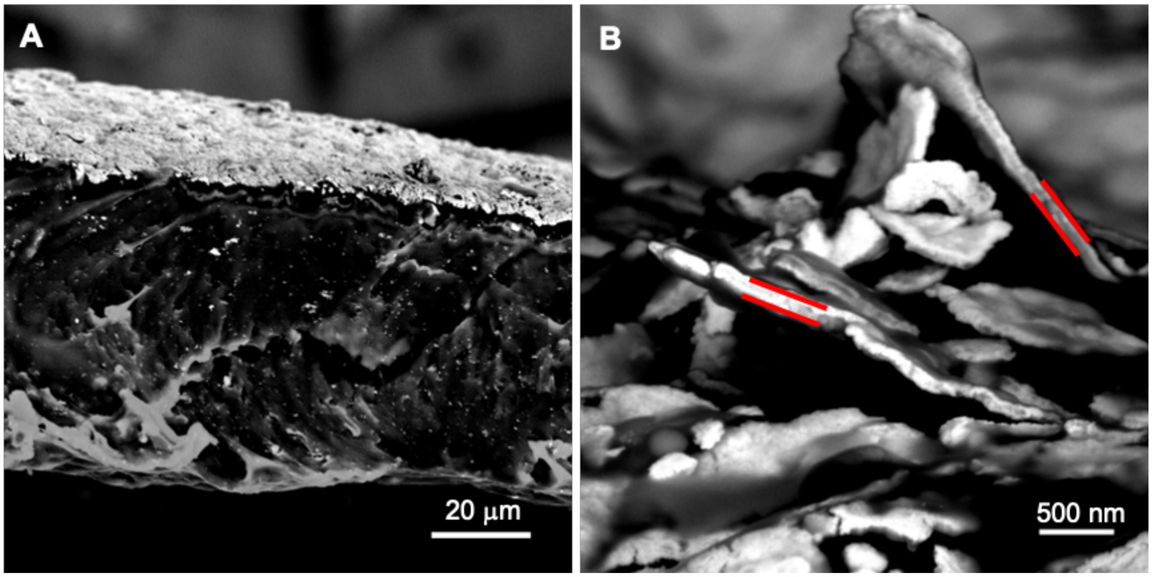

Figure S6. Cross sectional SEM of **(A)** an ENIG film on NBR, and **(B)** a close up of gold flakes at the cross-section edge. Red lines indicate width of the gold film. Related to Figure 2.

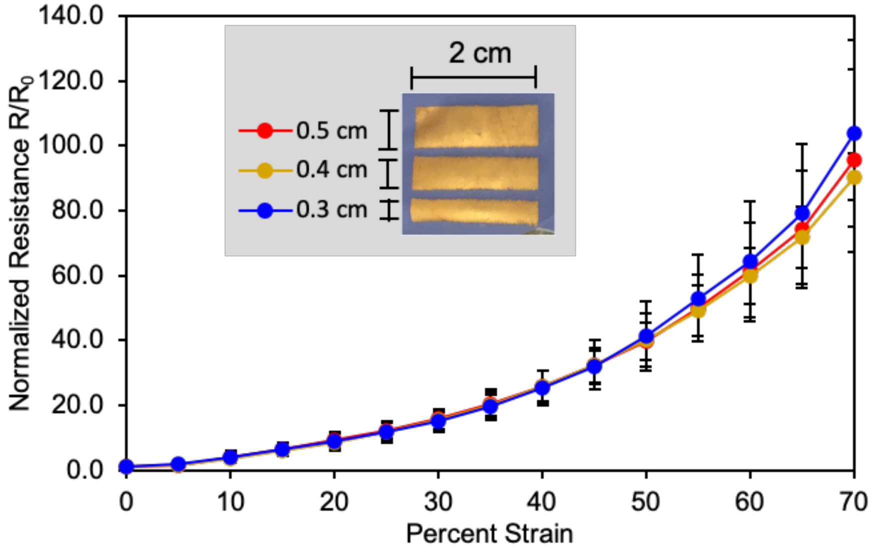

Figure S7. Resistance vs. percent strain for Au/NBR sensors that are 2 cm in length and 0.5 cm (red), 0.4 cm (yellow), and 0.3 cm (blue) in width. Related to Figure 3.

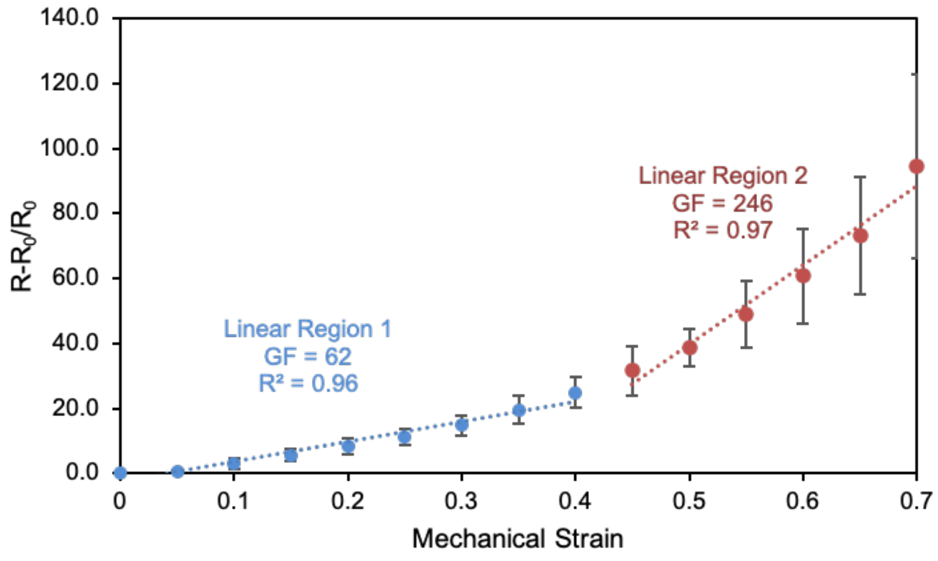

Figure S8. Linear fits of normalized resistance vs mechanical strain for Au/NBR sensors that are 2 cm in length and 0.5 cm in width. Related to Figure 3.

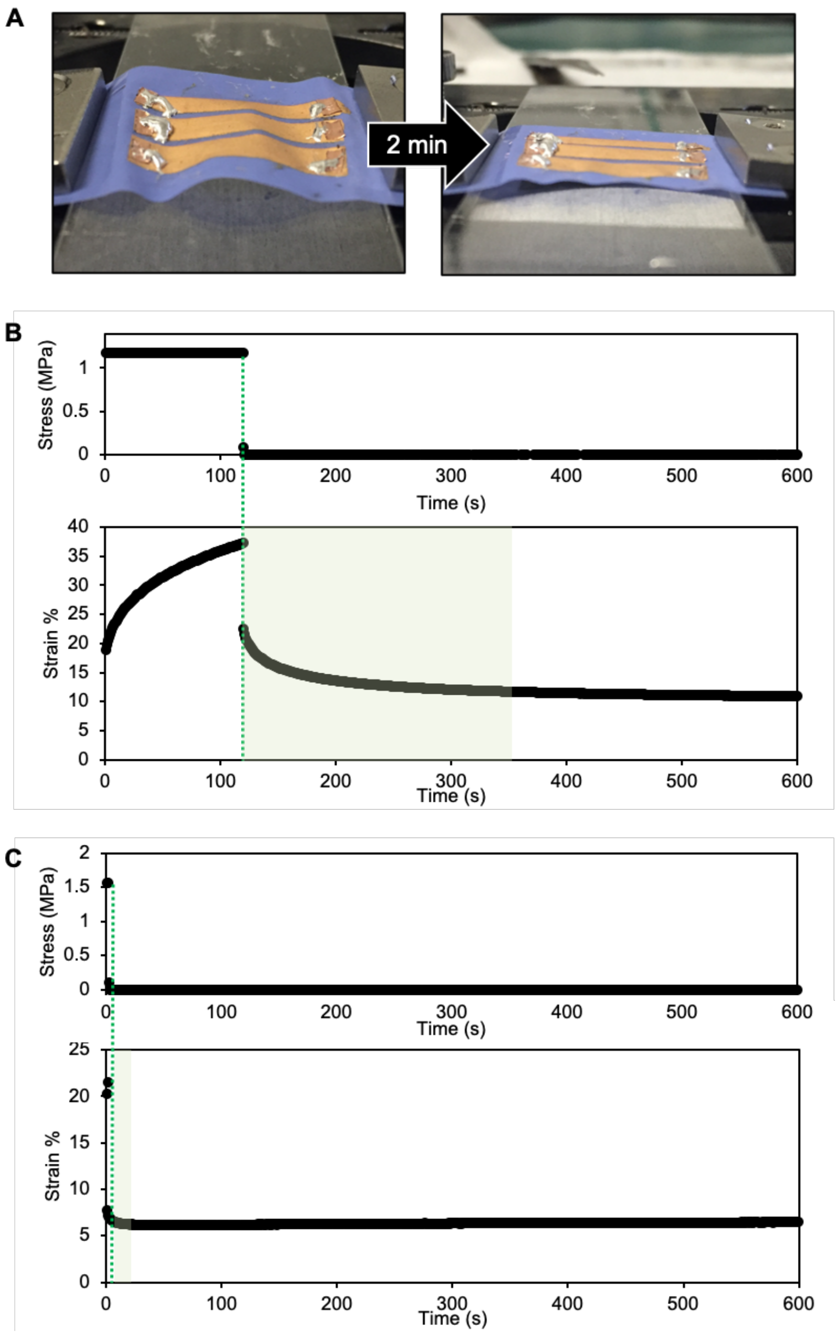

Figure S9. Strain recovery behavior of NBR. **(A)** Photographs of ENIG/NBR sensors immediately after relaxation from 40% to 0% and 2 minutes after the relaxation. **(B)** Dynamic mechanical analysis of the application of ~1.3 MPa stress for 2 minutes and recovery for 8 minutes. **(C)** Dynamic mechanical analysis of the application of ~1.3 MPa stress for 2 seconds and recovery for 8 minutes. Dotted lines indicate the time at which fixed stress was removed. Green boxes highlight the relaxation time to get to 90% of the final strain. Related to Figure 3 and Figure 4.

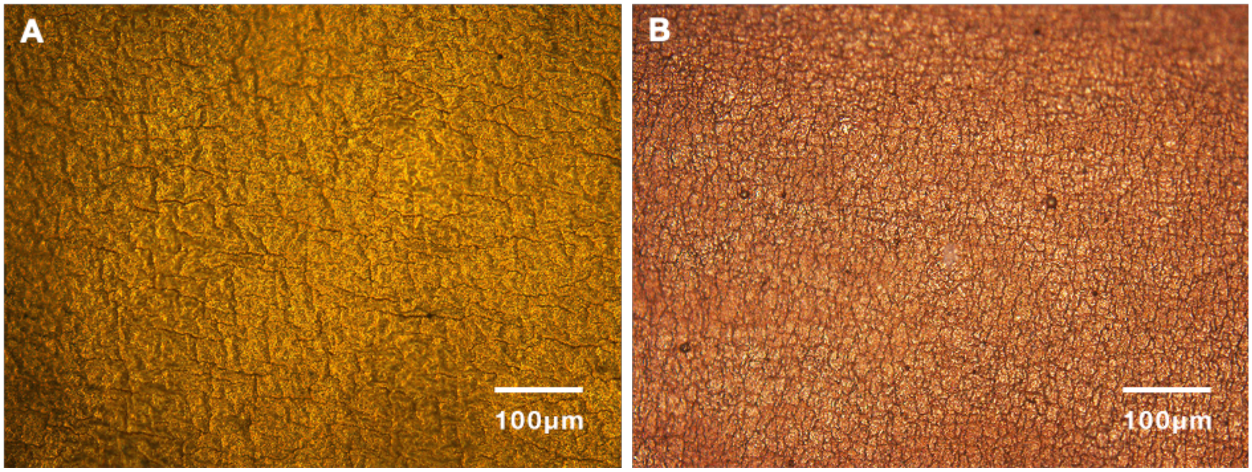

Figure S10. Optical micrographs of 0.5 mm-wide gold sensors on NBR after **(A)** one, and **(B)** 200 cycles of 40% strain. Related to Figure 3.

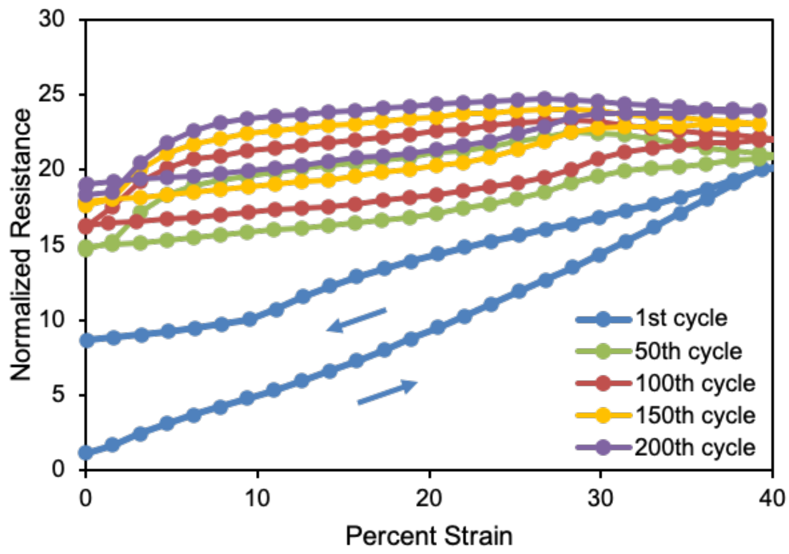

Figure S11. Hysteresis of the ENIG/NBR sensors during 200 cycles of 40% strain with no relaxation period between cycles. Related to Figure 3.

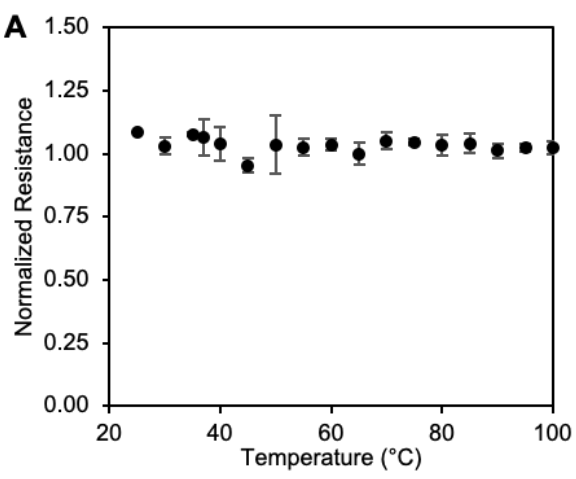

Figure S12. High-temperature behavior of NBR. **(A)** The change in resistance of the ENIG/NBR sensor with increasing temperature. **(B)** The change in resistance of the ENIG/NBR sensor with stretching at 100 °C. Related to Figure 3.

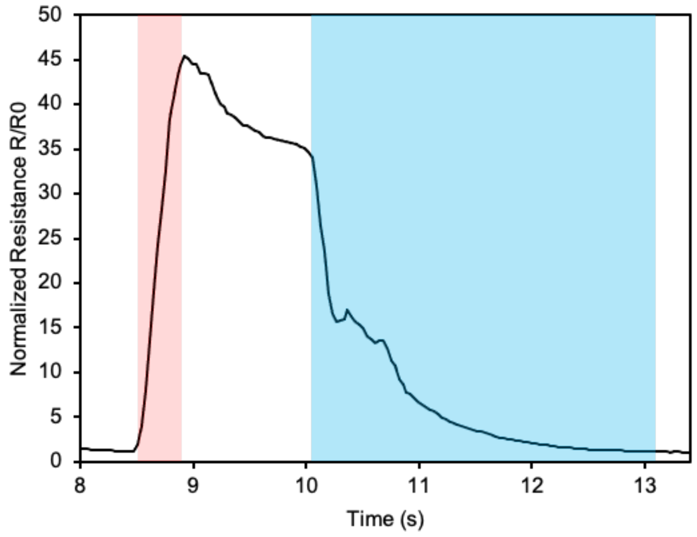

Figure S13. Normalized resistance of a PIP sensor with time as the wearer of the sensing glove closes and opens their hand. Response time is highlighted in red and recovery time is highlighted in blue. Related to Figure 4.

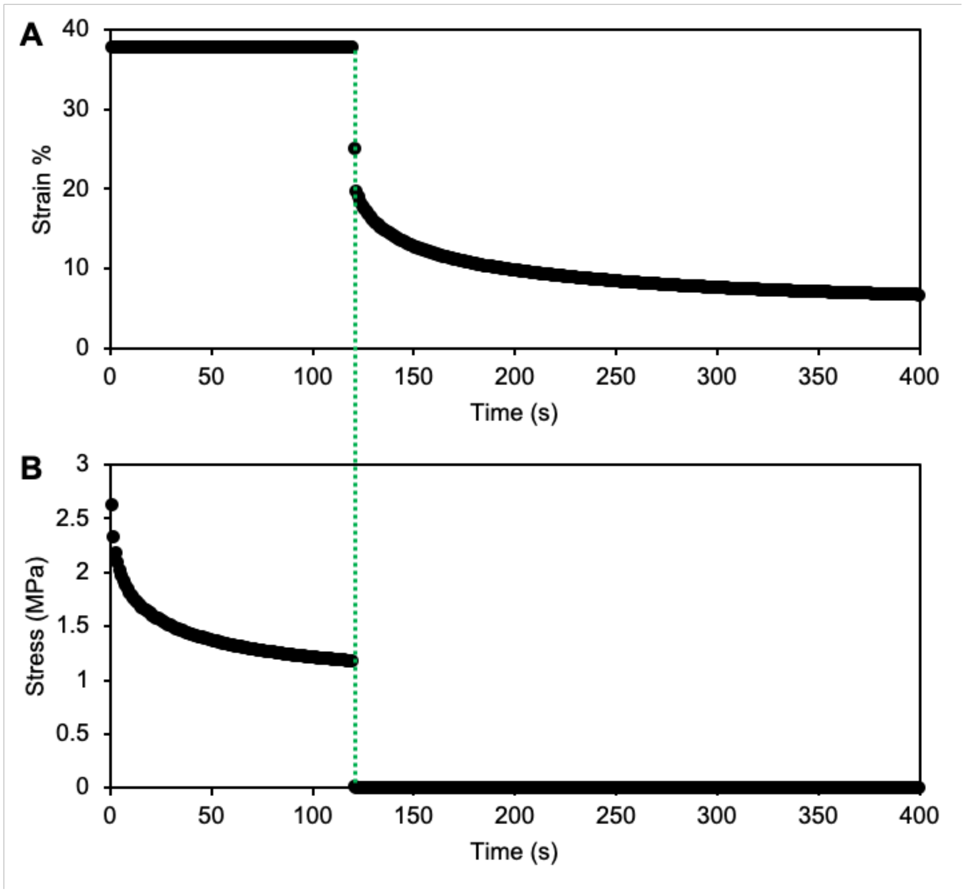

Figure S14. Stress relaxation behavior of NBR. **(A)** The application of ~40% strain and recovery. **(B)** The corresponding stress relaxation and recovery. Dotted green line indicates time when strain was removed. Related to Figure 4.

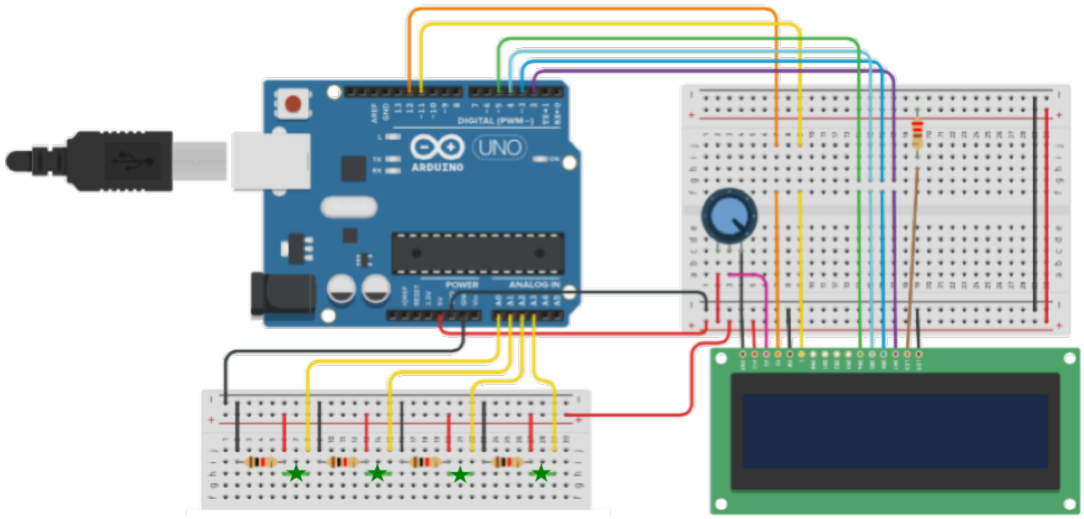

Figure S15. Schematic of the circuit used to display letters on the LCD screen. Green wires with stars indicate where a sensor can be connected to the circuit. Related to Figure 4 and STAR methods.

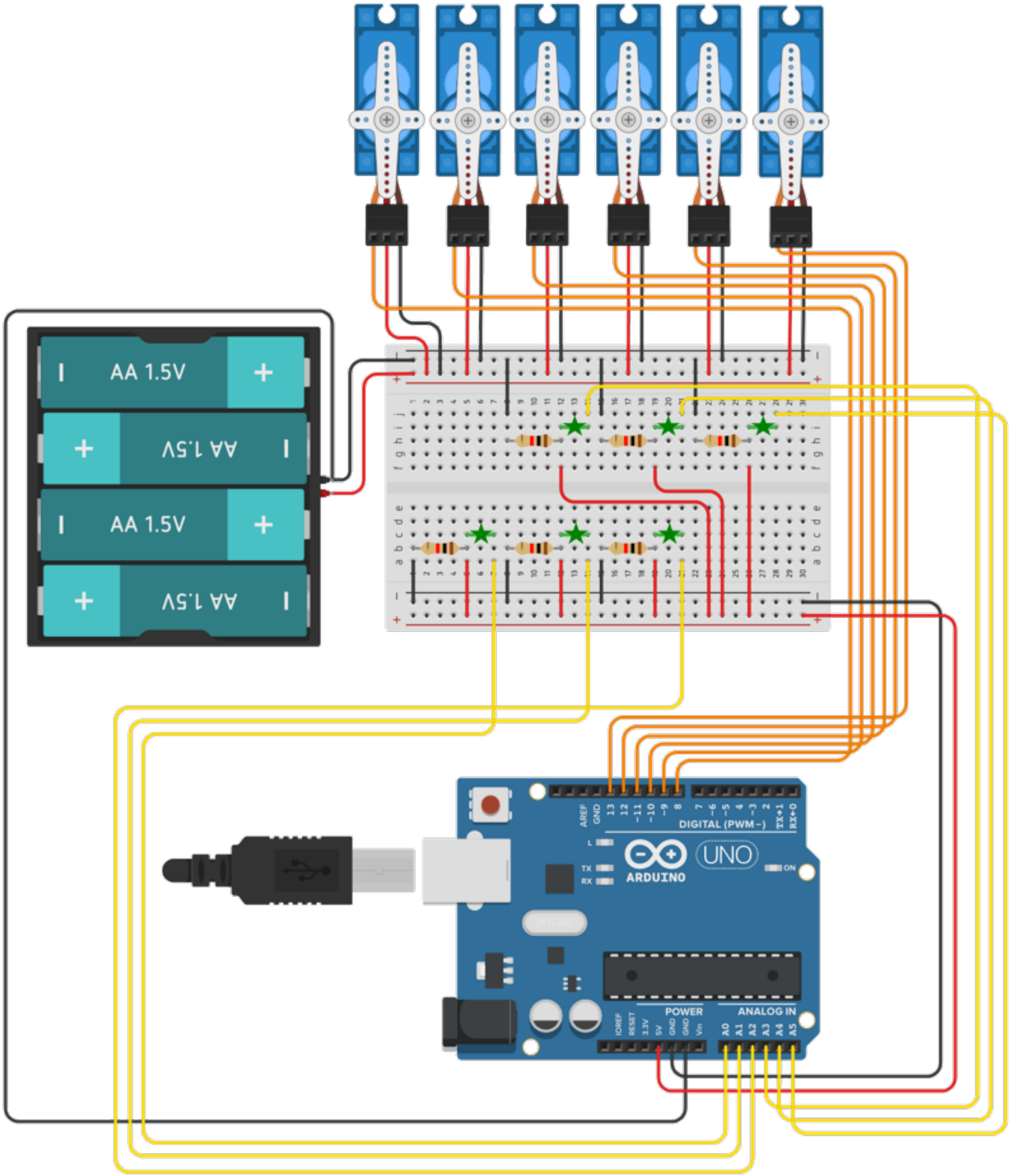

Figure S16. Schematic of the circuit used to control servo motor angles using finger gestures. Green wires with stars indicate where a sensor can be connected to the circuit. Related to Figure 4 and STAR methods.

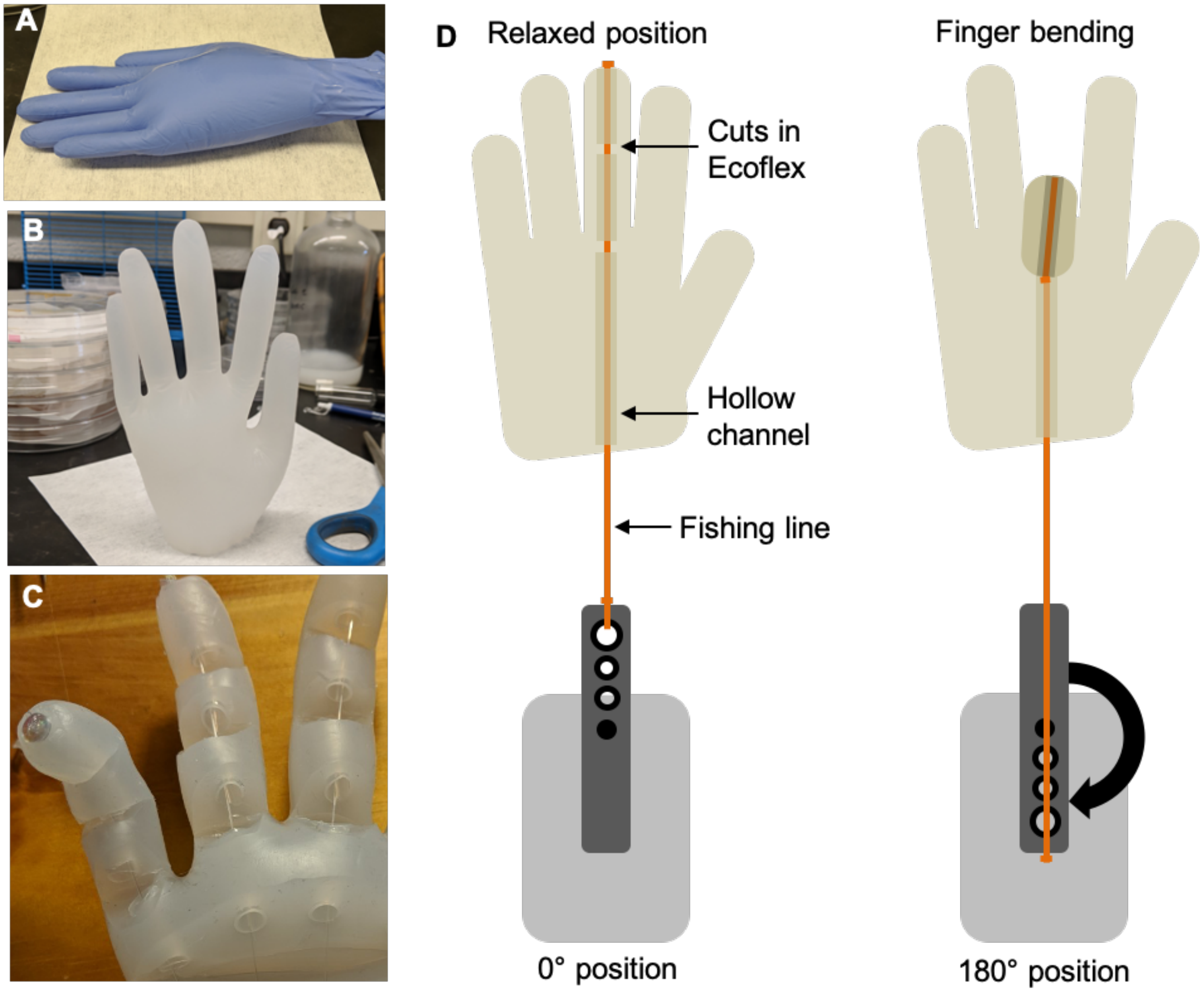

Figure S17. Fabrication of the Ecoflex robotic hand and the controlled bending actuation using servo motors. Photos of **(A)** an NBR glove filled with Ecoflex; **(B)** the Ecoflex hand model after curing and removing the NBR glove; **(C)** a close up of the robotic hand after channels are hollowed, lined, and fishing wire is threaded through each finger. **(D)** A schematic showing how bending actuation is achieved by controlling the servo motor position. Related to Figure 4 and STAR methods.

Table S1. Peak assignments corresponding to FT-IR spectra of NBR with APTES functionalization before and after POMA treatment. Related to Figure S2 and STAR methods.

Peak label in Fig. S2b, c	Wavelength (cm^{-1})	Vibration	Reference
1	3484	O-H stretch	(Celina et al., 1998; Gunasekaran et al., 2007; Kawashima and Ogawa, 2005)
2a,b	2923, 2852	C-H stretch	(Gunasekaran et al., 2007; Kawashima and Ogawa, 2005)
3	1779	C=O stretch (COOH)	(Zhou et al., 2007)
4	1714	C=O stretch (amide I)	(Miller et al., 2008)
5	1573	N-H bend (amide II)	(Miller et al., 2008)
6	1222	C-O stretch (COOH)	(Zhou et al., 2007)
